# *CFH* and *CFHR* structural variants in atypical Hemolytic Uremic Syndrome: Prevalence, genomic characterization and impact on outcome

**DOI:** 10.3389/fimmu.2022.1011580

**Published:** 2023-01-30

**Authors:** Rossella Piras, Elisabetta Valoti, Marta Alberti, Elena Bresin, Caterina Mele, Matteo Breno, Lucia Liguori, Roberta Donadelli, Miriam Rigoldi, Ariela Benigni, Giuseppe Remuzzi, Marina Noris

**Affiliations:** Istituto di Ricerche Farmacologiche Mario Negri IRCCS, Clinical Research Center for Rare Diseases Aldo e Cele Daccò and Centro Anna Maria Astori, Science and Technology Park Kilometro Rosso, Bergamo, Italy

**Keywords:** atypical hemolytic uremic syndrome (aHUS), eculizumab, factor H (FH), factor H-related proteins (FHRs), complement, copy number variations (CNVs), structural variants (SVs), single molecule real-time (SMRT)

## Abstract

**Introduction:**

Atypical hemolytic uremic syndrome (aHUS) is a rare disease that manifests with microangiopathic hemolytic anemia, thrombocytopenia, and acute renal failure, and is associated with dysregulation of the alternative complement pathway. The chromosomal region including *CFH* and *CFHR1-5* is rich in repeated sequences, favoring genomic rearrangements that have been reported in several patients with aHUS. However, there are limited data on the prevalence of uncommon *CFH-CFHR* genomic rearrangements in aHUS and their impact on disease onset and outcomes.

**Methods:**

In this study, we report the results of *CFH-CFHR* Copy Number Variation (CNV) analysis and the characterization of resulting structural variants (SVs) in a large cohort of patients, including 258 patients with primary aHUS and 92 with secondary forms.

**Results:**

We found uncommon SVs in 8% of patients with primary aHUS: 70% carried rearrangements involving *CFH* alone or *CFH* and *CFHR* (group A; n=14), while 30% exhibited rearrangements including only *CFHRs* (group B; n=6). In group A, 6 patients presented *CFH::CFHR1* hybrid genes, 7 patients carried duplications in the *CFH-CFHR* region that resulted either in the substitution of the last *CFHR1* exon(s) with those of *CFH* (*CFHR1::CFH* reverse hybrid gene) or in an internal *CFH* duplication. In group A, the large majority of aHUS acute episodes not treated with eculizumab (12/13) resulted in chronic ESRD; in contrast, anti-complement therapy induced remission in 4/4 acute episodes. aHUS relapse occurred in 6/7 grafts without eculizumab prophylaxis and in 0/3 grafts with eculizumab prophylaxis. In group B, 5 subjects had the *CFHR3_1-5_::CFHR4_10_* hybrid gene and one had 4 copies of *CFHR1* and *CFHR4*. Compared with group A, patients in group B exhibited a higher prevalence of additional complement abnormalities and earlier disease onset. However, 4/6 patients in this group underwent complete remission without eculizumab treatment. In secondary forms we identified uncommon SVs in 2 out of 92 patients: the *CFHR3_1-5_::CFHR4_10_* hybrid and a new internal duplication of *CFH*.

**Discussion:**

In conclusion, these data highlight that uncommon *CFH-CFHR* SVs are frequent in primary aHUS and quite rare in secondary forms. Notably, genomic rearrangements involving the *CFH* are associated with a poor prognosis but carriers respond to anti-complement therapy.

## Introduction

Atypical hemolytic uremic syndrome (aHUS) is an ultra-rare kidney disease characterized by microangiopathic hemolytic anemia, thrombocytopenia, and renal impairment (1). Primary aHUS is associated with genetic and acquired defects that led to dysregulation of the alternative pathway (AP) of complement system, resulting in endothelial damage in the microcirculation of the kidney and other organs (2). About 50% of patients carry genetic abnormalities that affect genes coding for complement regulators (*CFH*, *CD46*, *CFI* and *THBD*) and components (*C3* and *CFB*), while in 10% of patients anti-FH autoantibodies have been reported (3).

Atypical HUS can be secondary to autoimmune or systemic disease, pregnancy/postpartum, malignant hypertension, drug treatments, cancer and transplantation (4, 5). In the secondary forms, the prevalence of genetic defects is variable, ranging from almost 60% in cases associated with malignant hypertension or pregnancy to less than 10% in drug-induced TMA.

**Figure 1:**
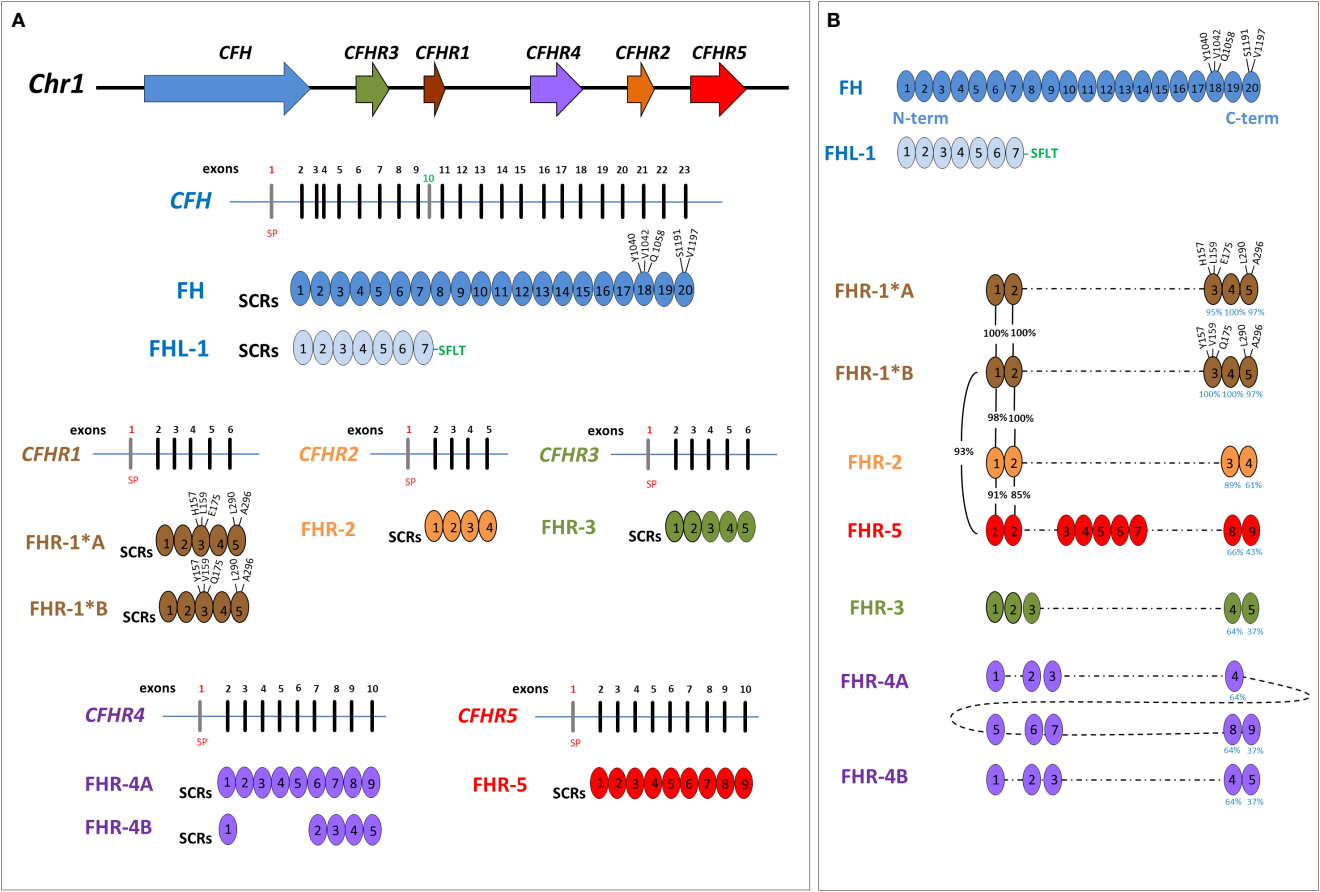
Structure of Factor H family: genes and proteins. **(A)** The human complement factor H (*CFH*) gene family is located on chromosome 1q31.3 and includes six genes: *CFH, CFHR3, CFHR1, CFHR4, CFHR2* and *CFHR5*. For each gene, the corresponding protein was represented. Each short consensus repeat (SCR) is composed of about 60 amino acids and is encoded by a single exon, with the exception of SCR2 of Factor H (FH), encoded by exon 3 and 4. Exon 1 of each gene encodes 18 amino acids of the signal peptide (SP). *CFH* gene is composed of 23 exons and, through two alternative splicing, produces FH, deriving from 22 exons, and Factor H-like protein 1 (FHL-1), deriving from 10 exons. Exon 10 is not included in the FH transcript and encodes the C-terminal four amino acids (Ser-Phe-Leu-Thr; indicated in the Figure with the green SFLT) and the 3’UTR of FHL-1. FHR-1 exists in two isoforms that differ in three amino acids in the SCR3: FHR-1*A is known as acidic isoform and has His at position 157 (H157), Leu at 159 (L159) and Glu at 175 (E175); FHR-1*B is the basic isoform with Tyr at position 157 (Y157), Val at 159 (V159) and Gln at 175 (Q175). **(B)** Factor H-related proteins (FHRs) share a high degree of conservation within the C-terminal domains of FH (SCR18-SCR19-SCR20), and FHR-1 is the most similar (the percentage of amino acids identity between each SCR of FHR and those of FH is indicated by blue numbers under the SCRs). As represented in the Figure, SCR3 of FHR-1*A differs from SCR18 of FH for 3 amino acids (H157, L159 and E175) while SCR5 differs from FH-SCR20 for 2 amino acids (L290 and A296). At variance, SCR3 of FHR-1*B has the same amino acids of SCR18 of FH. Of note, the N-terminal domains of FHR-1, FHR-2 and FHR-5 (SCR1 and SCR2) have a high sequence identity (indicated by black percentage numbers) and include a dimerization motif which explains their presence in plasma as either homo-or heterodimers. This image was inspired by Jozsi et al. (7) Trends immunology, 2015.

The gene most commonly involved in aHUS is *CFH*, encoding complement factor H (FH). *CFH* is mapped on chromosome 1q31 within the RCA (Regulation of Complement Activation) gene cluster, which also includes the *CFHR3*, *CFHR1, CFHR4, CFHR2* and *CFHR5* genes, derived from genomic duplication events (6). The resulting FH and FHR proteins are organized in short consensus repeats (SCRs), each consisting of about 60 amino acids (**Figure 1**), and are mainly produced by the liver and circulate in the blood.

Factor H is the main plasma regulator of the AP of complement and consists of 20 SCRs. FH regulatory activity is mediated by its N-terminal domains (SCR1-4) acting as a cofactor for complement protease Factor I (FI) and accelerating the decay of C3 convertase. In addition, through the SCR6-7 and C-terminal domains (SCR19-20), FH binds C3b and polyanions, such as glycosaminoglycans, heparan sulfate, and sialic acids, and mediates cell surface protection from complement activation. The large majority of *CFH* genetic abnormalities in aHUS cluster in the C-terminal part of the protein, leading to reduced complement regulation on endothelial cells. However, not all the carriers of heterozygous *CFH* genetic defects manifest aHUS, due to incomplete penetrance.

FHRs were originally thought to be negative complement inhibitors, but later studies indicated that these molecules may instead enhance complement activation (8). The C-terminal domains of FHRs have a high level of amino acidic sequence identity with SCR18-19-20 of FH leading them to be able to bind the same FH ligands (**Figure 1**) (8–11). The N-terminal SCRs (SCR1-2) of FHR-1, FHR-2 and FHR-5, are very similar (85-100%) and include a dimerization domain (**Figure 1**), which explains their presence in the circulation as homo- and hetero- dimers or tetramers (12–14). This oligomerization increases FHR avidity for C3b and C3-opsonized surfaces and for polyanionic surface ligands, which results in the activation of the alternative pathway (12, 13). The N-terminal domains of FHR-3 and FHR-4 (SCR1-3 of both) share high residue sequence similarity with FH SCR6 to SCR8 and FH SCR6-8-9, respectively, which are involved in binding to heparin, C-reactive protein, and microbial surface ligands. However, none of the FHR protein domains have any similarity to the N-terminal regulatory domains of FH, indicating that FHRs lack direct complement regulatory activity, although this point remains controversial (15).

The FH gene cluster is characterized by large repeated regions, which favors genomic rearrangements and copy number variations (CNVs) like the duplications, deletions and inversions that have been reported in association with aHUS and other complement-mediated diseases (16, 17). Genomic alterations involving DNA segments larger than 1kb are defined as structural variants (SVs) and the most frequent SV in the *CFH* gene cluster is the ~84 kb deletion of *CFHR3* and *CFHR1* (*CFHR3-CFHR1* del), which is associated with a high risk of developing anti-FH autoantibody (anti-FHs)-mediated aHUS (18). Rare SVs involving *CFH* and *CFHRs* that lead to hybrid genes such as *CFH::CFHR1, CFH::CFHR3*, and the reverse *CFHR1::CFH* hybrids have been reported in patients with primary aHUS, and a few functional analyses have confirmed their involvement in disease pathogenesis (7, 16, 17).

However, data on the prevalence of *CFH-CFHR* genomic rearrangements in primary and secondary aHUS, their impact on disease penetrance, disease onset, response to therapy and outcome are limited to case reports or case-series.

Here, we report a retrospective study of *CFH-CFHR* copy number variations (CNVs) in a large cohort of unrelated patients affected by primary (n=258) or secondary aHUS (n=92). We evaluated the prognosis of patients carrying *CFH-CFHR* SVs and the contribution of the concomitant presence of rare complement gene variants or anti-FHs abnormalities to disease development. To overcome the limits of next generation sequencing (NGS) to detect SVs in the *CFH-CFHR* region, we applied Multiplex Ligation-dependent Probe Amplification (MLPA), long-read sequencing (Single -Molecule Real-Time, SMRT) and direct sequencing to identify and characterize rare genomic rearrangements. We found them in the 6% of patients, including 2 patients with secondary forms. Furthermore, we identified a group of patients carrying rearrangements that included only *CFHR* genes, which have so far been reported in association with C3G or Immune complex-mediated membranoproliferative glomerulonephritis (IC-MPGN) (17). This group presented a milder disease phenotype than patients with rearrangements involving *CFH*.

Our results confirm the important role of *CFH* genomic rearrangements in the pathogenesis of aHUS and highlight the potential impact of SVs involving *CFHR* genes in disease predisposition and phenotype.

## Material and methods

### Study participants

Patients included in this study were recruited through the International Registry of HUS/TTP, under the coordination of the Aldo and Cele Daccò Clinical Research Center for Rare Diseases (Ranica, Bergamo, Italy).

Clinical information and demographic/laboratory data for patients and their available relatives were collected using a case report form. Biochemical and genetic tests were performed using blood, plasma or serum samples, and DNA was collected for each patient and available relatives.

Healthy controls were recruited among blood donors and were analyzed for copy number variations (CNVs). The samples used for the research were stored at the Centro Risorse Biologiche (CRB) Mario Negri, Malattie Rare e Malattie Renali biobank.

Atypical HUS was diagnosed in all cases with microangiopathic hemolytic anemia and thrombocytopenia (hematocrit less than 30%, hemoglobin level less than 10 g/dL, serum lactate dehydrogenase level higher than 500 U/L, undetectable haptoglobin, fragmented erythrocytes in peripheral blood smear, and platelet count less than 150x10^3^/µl) associated with acute renal failure (serum creatinine>1.3 mg/dl for adults, >0.5 mg/dl for children under 5 years of age and >0.8 mg/dl for children aged 5-10 years old; and/or urinary protein/creatinine ratio >200 mg/g; or an increase of serum creatinine or urinary protein/creatinine ratio>15% compared to baseline levels). Thrombotic thrombocytopenic purpura was ruled out in the presence of ADAMTS13 activity >10% and no anti-ADAMTS13 antibodies. Patients were classified as having primary aHUS when both secondary underlying conditions and Stx-*E.Coli* infections were ruled out; a secondary form was considered when aHUS was associated with hypertension, autoimmune diseases, infections, pre-existing nephropathy, transplantation, drug exposure or other coexisting conditions (pneumococcal infections and malignancy).

Familial aHUS was diagnosed when two or more members of the same family were affected by the disease at least 6 months apart and exposure to a common trigger infectious agent was excluded. Sporadic aHUS was diagnosed when one or more episodes of the disease manifested in a subject with no familial history of the disease.

The study was approved by the Ethics Committee of the Azienda Sanitaria Locale, Bergamo (Italy) and informed consent was obtained in accordance with the Declaration of Helsinki.

### Complement profile assessment

FH and anti-FH autoantibody serum levels were measured using Enzyme-Linked Immunosorbent Assay (ELISA) as previously reported (3).

### Genetic screening and biochemical testing

Genomic DNA was extracted from peripheral blood leukocytes (Nucleon™ BACC2 kit, GE Healthcare; NucleoSpin Blood columns, Macherey-Nagel). All coding exons and the intronic flanking regions of membrane cofactor protein (*CD46)*, complement factor H (*CFH*), complement factor I (*CFI)*, complement factor B (*CFB)*, complement C3 (*C3)* and thrombomodulin (*THBD)* genes were amplified by polymerase chain reaction (PCR) and were directly sequenced (48-capillary 3730 DNA Analyzer), as previously reported (19). Patients recruited more recently were analyzed using a next generation sequencing (NGS) panel for the simultaneous sequencing of *CFH*, *CD46*, *CFI*, *CFB, C3*, and *THDB* through Ion Torrent platform (Life technologies). Since recessive LPVs in the gene encoding diacylglycerol kinase DGKε (*DGKE)* have been identified in patients with aHUS with an onset in infancy, we also sequenced *DGKE*, by NGS, in patients carrying uncommon SVs and with a disease onset below 4 years (20, 21).

Patients with uncommon SVs and their available relatives, were genotyped by NGS or direct sequencing for the *CFH* and *CD46* single-nucleotide polymorphisms (SNPs) that define the aHUS-risk haplotypes *CFH-H3* and *CD46_GGAAC_*, respectively (19, 22–25).

Genetic variants with a reported minor allelic frequency (MAF) below 0.001 in the Genome Aggregation Database (gnomAD) and with a Combined Annotation Dependent Depletion (CADD) phred score ≥10 were considered likely pathogenic variants (LPVs).

### *CFH*-*CFHR* copy number variations

Multiplex ligation dependent probe amplification (SALSA MLPA P236, MRC Holland, Netherlands) and in-house probes for *CFHR4* and *CFHR5* were used to evaluate the presence of copy number variations (CNVs) in *CFH*, *CFHR1*, *CFHR2*, *CFHR3*, *CFHR4*, and *CFHR5* genes in all the patients, as previously reported (17).

Two hundred and fourteen healthy subjects were also analyzed for *CFHR4* CNVs using multiplex polymerase chain reaction (mPCR) amplifying intron 1 and exon 2 of *CFHR4* and intron 3 of *CFHR1* (18).

### Single molecule real-time (SMRT) sequencing

Probes targeting *CFH-CFHRs* on the human genome, reference hg19 (from chr: 196619000 to chr: 196979303) were designed by Nimble Design Software (Roche Sequencing, Pleasanton, CA, US). Selected DNA samples previously identified with CNVs through MLPA analysis, were sequenced at the Norwegian Sequencing Centre using the PacBio Sequel system. Methodology details have previously been published in Piras R et al. (17)

### Direct sequencing

PCR was carried out in 25 µl of reaction volume using 125 ng of genomic DNA from patients with abnormal MLPA pattern and the Accuprime Taq DNA Polymerase (Thermo Fisher Scientific; 35 cycles of amplification: 94°C for 30 seconds, 59°C for 30 seconds, 68°C for 10 minutes). The breakpoint regions were identified by bidirectional sequencing of the long-range PCR product, using BigDye^®^ Terminator v3.1 Cycle Sequencing Kit (Thermo Fisher Scientific) following the manufacturer’s instructions. The BigDye XTerminator^®^ Purification Kit (Thermo Fisher Scientific) was used to purify DNA sequencing reactions removing non-incorporated BigDye^®^ terminators and salts. Sequencing analyses were carried out on the 48-capillary 3730 DNA Analyzer (Life Technologies). Sequences of primers used for long-range PCR are reported in **Supplementary Table 1**.

### Western blot

The molecular pattern of FH-FHRs was studied by Western Blot (WB) using serum/plasma (diluted 1:40 for FHRs and 1:80 for FH). Proteins were separated by 10–12% SDS-PAGE, under non-reducing conditions and transferred by electroblotting to polyvinylidene Difluoride (PVDF) membrane (Bio-Rad). Membranes were developed using specific FH/FHR antibodies (the FHR-3 polyclonal antiserum was a kind gift from Prof. Zipfel (15); the anti-FHR1-2-5 monoclonal antibody was kindly provided by Prof. de Cordoba (12); the commercial monoclonal anti-human Factor H - OX-23, LSBio-), followed by HRP- conjugated secondary antibodies and ECL chemiluminescence detection system (Amersham).

### Statistical analysis

All statistical tests were executed using MedCalc software. The Chi-square test or the Fisher’s exact test were used to make comparisons, as appropriate.

## Results

### *CFH-CFHR* structural variants in aHUS

We report a retrospective MLPA analysis in a cohort of 350 unrelated patients with a diagnosis of aHUS, including 258 with primary aHUS and 92 with secondary aHUS.

Common structural variants (SVs), namely the *CFHR3-CFHR1* deletion (*CFHR3-CFHR1*del) and/or the *CFHR1-CFHR4* deletion (*CFHR1-CFHR4*del), were observed in 165 patients (47%; **Table 1**). The homozygous *CFHR3-CFHR1*del was significantly more frequent in aHUS cases than in healthy controls (11% vs 3%, respectively, p-value = 0.01).

**Table 1:** Frequency of common and uncommon Structural Variants (SVs) in controls and patients.

	Controls	Total aHUS cases (n=350)		Primary aHUS (n=258)		Secondary aHUS (n=92)	
	n/tot (%)	n (%)	*P*-value^a^	n (%)	*P*-value^a^	n (%)	*P*-value^a^
Common SVs							
Het *CFHR3-CFHR1* del	32/100 (32%)	114 (32.6%)	ns	77 (29.8%)	ns	37 (40.2%)	ns
Hom *CFHR3-CFHR1*del	3/100 (3%)	40 (11.4%)	**0.01**	36 (14%)	**0.002**	4 (4.3%)	ns
Het *CFHR1-CFHR4*del	4/214 (1.9%)	2 (0.6%)	ns	1 (0.4%)	ns	1 (1.1%)	ns
*CFHR3-CFHR1*del + *CFHR1-CFHR4*del	0/214 (0%)	9 (2.6%)	ns	7 (2.7%)	*0.02*	2 (2.2%)	*0.09*
Uncommon SVs							
	1 (1%)*	22 (6%)	ns	20 (7.8%)	ns	2 (2.2%)	ns

a P-value have been calculated considering patients versus controls; Significant values are reported in bold; ns, not statistically significant.

*CFHR3_1-5_::CFHR4_10_ hybrid gene.

The prevalence of common SVs was comparable in primary and secondary aHUS patients (47% vs 48%, respectively, ns), although the homozygous *CFHR3-CFHR1*del was more frequent in primary than in secondary aHUS (14% vs 4%, respectively, p-value = 0.01).

Specifically, in the primary aHUS group, common SVs were detected in 121 patients (47%) (**Table 1**): the heterozygous *CFHR3-CFHR1*del was found in 77 patients (30% vs 32% in healthy controls, ns); 36 patients exhibited the homozygous *CFHR3-CFHR1*del (14% vs 3% controls, p-value = 0.002); in addition, 7 patients were carriers of both *CFHR3-CFHR1*del and *CFHR1-CFHR4*del (3% vs 0% controls, ns) and a single case had the heterozygous *CFHR1-CFHR4*del (0.4% vs 2% controls, ns).

Among patients with secondary aHUS, common SVs were detected in 44 patients (48%). As shown in **Table 1**, 37 patients were heterozygous for the *CFHR3-CFHR1*del (40% vs 32% controls, ns) and 4 patients exhibited the same deletion on both alleles (4% vs 3% controls, ns). In addition, 2 patients were carriers of both the *CFHR3-CFHR1*del and the *CFHR1-CFHR4*del (2% vs 0% controls, p-value = *0.09*) and one patient had the heterozygous *CFHR1-CFHR4*del (1% vs 2% controls, ns).

Twenty-two patients (6%) carried uncommon SVs, including new or rare duplications and hybrid genes. These uncommon SVs were mainly found in patients with primary aHUS (n=20 out of 22 carriers; 91%), with a prevalence in this group of 8%.

Seventy % are rearrangements involving *CFH* gene alone or *CFH* and *CFHR* genes (n=14) and 30% are rearrangements including only *CFHR* genes (n=6; **Table 2**) that were mainly reported in C3G (17).

**Table 2:** List of patients with new/rare *CFH-CFHR* structural variants (SVs) and/or other complement abnormalities.

Rare abnormalities							
Pat.	Uncommon SVs	LPVs	gnomAD MAF_all	CADD	FH levels (mg/L)	Anti-FH abs	g.A>G rs7542235 snp (*CFHR3-CFHR1*Δ tag)
Primary aHUS							
Group A							
#1	*CFH_1-21_::CFHR1_5-6_* hybrid gene + *de novo CFHR3-CFHR1* dupl				408	Neg	AA
#2	*CFH_1-21_::CFHR1_5-6_* hybrid gene				NA	NA	AA
#3	*CFH_1-21_::CFHR1_5-6_* hybrid gene	*CFH*: p.R1210C	2.00E-04	11.77	377	Neg	AA
#4 ^1^	*CFH_1-21_::CFHR1_5-6_* hybrid gene	*CFI*: c.1429+1G>C	2.80E-05	24.7	NA	NA	AA
#5	*CFH_1-22_::CFHR1_6_* hybrid gene				NA	Neg	AA
#6	*CFH_1-20_::CFHR1_4-5-6_* hybrid gene + *CFHR1*dupl				327	Neg	AA
#7	Reverse *CFHR1_1-5_::CFH_23_* hybrid gene				332	Neg	AG
#8 ^2^	Reverse *CFHR1_1-5_::CFH_23_* hybrid gene+ *CFHR3* dupl				218	Neg	AA
#9	Reverse *CFHR1_1-5_::CFH_23_* hybrid gene				363	NA	AG
#10	Reverse *CFHR1_1-4_::CFH_22-23_* hybrid gene + *CFHR3* dupl				182	Neg	AA
#11	Reverse *CFHR1_1-4_::CFH_22-23_* hybrid gene				221	Neg	AG
#12	Reverse *CFHR1_1-4_::CFH_22-23_* hybrid gene				283	Neg	AA
#13	*CFH_1-18_* duplication				210	Neg	AA
#14	Reverse hybrid *CFHR1*_1-3_::*CFH*_21-23_ gene + *CFHR3_1-3_* dupl				327	Neg	AA
Group B							
#15	*CFHR3_1-5_::CFHR4_10_* hybrid gene	*C3*: p.D1115H	0	27.4	304	Neg	AA
#16	*CFHR3_1-5_::CFHR4_10_* hybrid gene				308	Pos	GG
#17	*CFHR3_1-5_::CFHR4_10_* hybrid gene				376	Neg	AA
#18	*CFHR3_1-5_::CFHR4_10_* hybrid gene	*CD46*: c.286+2T>G	5.21E-05	14.54	NA	NA	GG
#19	*CFHR3_1-5_::CFHR4_10_* hybrid gene				233	Neg	AA
#20 ^3^	*CFHR1-CFHR4* duplication (4 copies)	*GRHPR:* p.R96H (hom)	3.98E-06	16.1	316	Neg	AA
Secondary aHUS							
#21	*CFHR3_1-5_::CFHR4_10_* hybrid gene				388	Neg	AA
#22	*CFH_2-9_* duplication				NA	NA	AA

Abbreviations and limits of normal range:

SVs, structural variants defined as genomic rearrangements resulting in duplications, deletions and inversions larger than 1 kb;

NA, not available;

Normal serum/plasma FH levels: ≥193 mg/L;

LPV, likely pathogenic variants, defined as genetic variants in coding and splicing regions of complement genes (*CFH, CFI, CD46, CFB, C3,* and *THBD*) with MAF< 0.001 in the gnomAD Database and with a CADD phred score ≥10;

^1^Bresin et al. (26);^2^ Valoti et al. (27);^3^Valoti et al. (28).

From here on we divided patients with rearrangements in *CFH* alone or *CFH* and *CFHR* genes (group A) from those involving only *CFHRs* (group B) to investigate differences in prevalence, age of onset, outcome and response to therapy.

### Primary aHUS group A

**Figure 2:**
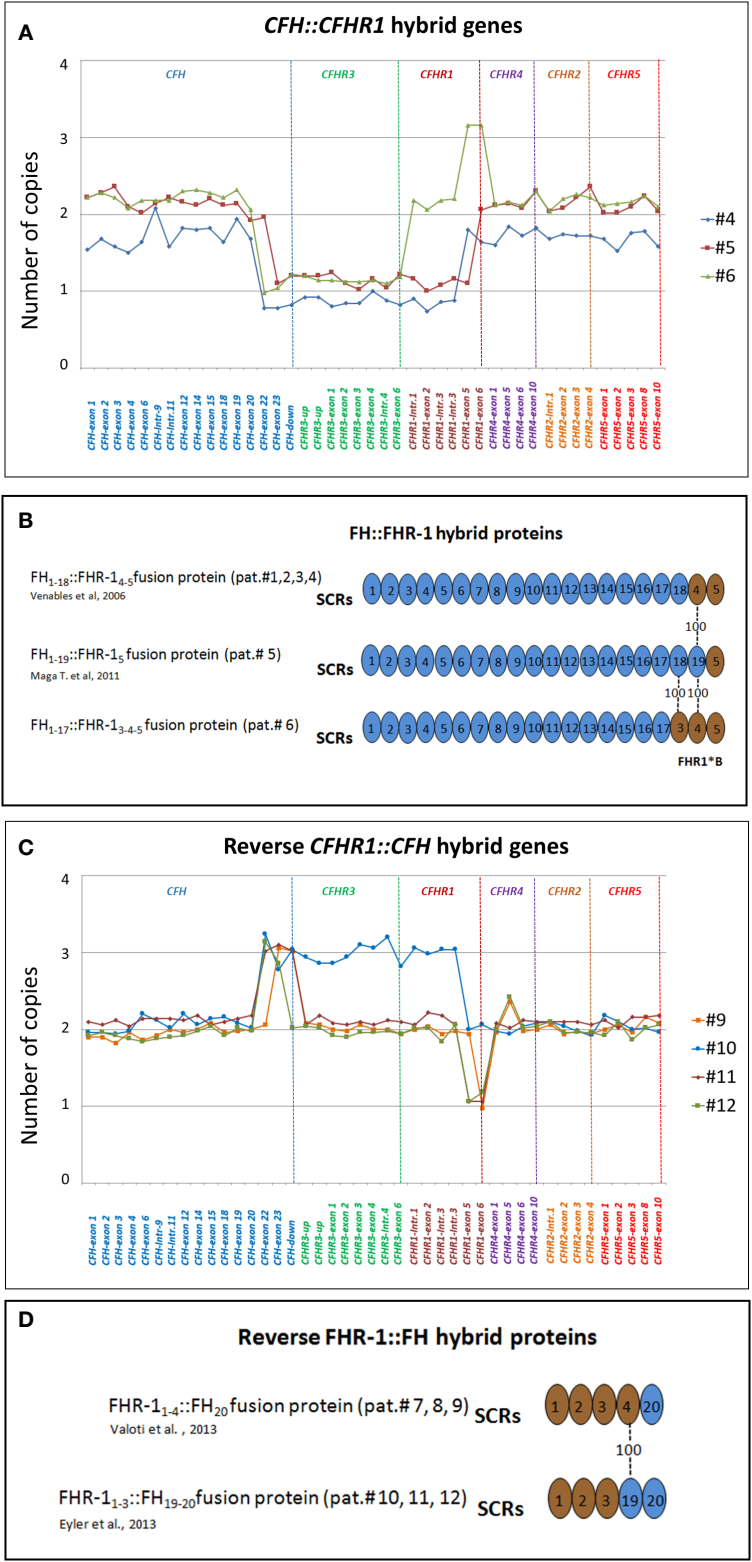
Graphic representation of MLPA results from aHUS patients carrying structural variants (SVs) in the *CFH* and *CFHR* genes. **(A)** MLPA patterns consistent with *CFH::CFHR1* hybrid genes. In patient #4, MLPA pattern shows one copy of exons 22 and 23 of *CFH*, one copy of *CFHR3*, one copy of *CFHR1* until intron 3, 2 copies of exons 5 and 6 of *CFHR1* and two normal copies of *CFHR4, CFHR2* and *CFHR5*, consistent with *CFH_1-21_::CFHR1_5-6_* hybrid gene (29). In patient #5 MLPA results evidence 1 copy of exon 23 of *CFH*, one copy of *CFHR3*, one copy of *CFHR1* until exon 5, 2 copies of exons 6 of *CFHR1* and two normal copies of *CFHR4, CFHR2* and *CFHR5*, consistent with *CFH_1-22_::CFHR1_6_* hybrid gene (30). In patient #6 MLPA analysis shows one copy of exons 22 and 23 of *CFH*, one copy of *CFHR3*, normal copy number from the *CFHR1-*intron 1 to *CFHR1-*intron 3, 3 copies of exons 5 and 6 of *CFHR1*, and two normal copies of *CFHR4, CFHR2* and *CFHR5.* This abnormal MLPA pattern was further characterized through long PCR, Sanger sequencing and SMRT which, as reported in **Figure 3**, led to the identification of the *CFH_1-20_::CFHR1_4-6_* hybrid gene. **(B)** Representation of FH::FHR-1 hybrid proteins resulting from SVs identified in patients #1, #2, #3, #4, #5 and #6. SCRs translated from *CFH* are indicated in blue while SCRs deriving from *CFHR1* are indicated in brown. The number “100” indicates that SCR4 of FHR-1 is identical to SCR19 of FH; similarly, SCR3 of FHR-1*B is identical to SCR18 of FH. The total identity between FH and FHR-1 indicates that the translated FH::FHR-1 protein is the same in all the above described cases. **(C)** MLPA pattern consistent with reverse *CFHR1::CFH* hybrid genes. MLPA results in patient #9 show 3 copies of *CFH*-exon 23, normal *CFHR3* copies, two *CFHR1* copies until exon 5, one copy of *CFHR1*-exon 6, two normal copies of *CFHR4, CFHR2* and *CFHR5*, consistent with the reverse *CFHR1_1-5_::CFH_23_* hybrid gene. In patient #10, #11 and #12 MLPA analysis shows a gain starting from *CFH-*exon 22 until *CFHR1-*intron 3 and a loss of *CFHR1*-exon 5-6 consistent with a reverse *CFHR1_1-4_::CFH_22-23_* hybrid gene. In addition, patient #10 carries 3 copies of *CFHR3* and 2 copies of normal *CFHR1.* In patient #11 MLPA provides a copy of normal *CFHR1* and 2 copies of *CFHR3*, consistent with the presence of *CFHR1_1-4_-CFH_22-23_* on one allele and *CFHR3-CFHR1 del* on the other allele (see **Supplementary Figure 1**). Unlike patient #11, the abnormal MLPA pattern of patient #12 does not involve the probe located downstream of *CFH*. **(D)** Representation of reverse FHR-1::FH hybrid proteins resulting from SVs identified in patients #7, #8, #9, #10, #11 and #12. The translated fusion protein is the same in all cases due to the 100% of identity between SCR19-FH and SCR4-FHR-1.

**Figure 3:**
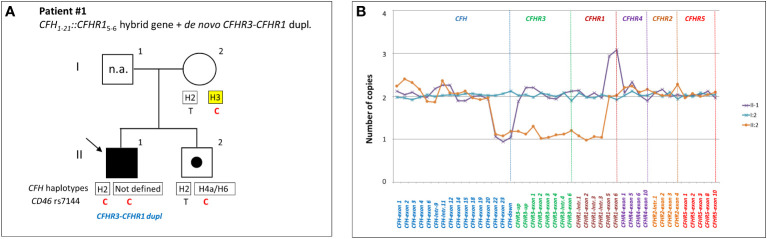
Patient #1 with a hybrid *CFH_1-21_::CFHR1*_5-6_ gene and a *de novo CFHR3-CFHR1* duplication. **(A)** The proband (black arrow) is patient II:1, his father is I:1 (samples are not available, n.a.), his mother is I:2 and his brother (unaffected carrier of hybrid *CFH_1-21_::CFHR1*_5-6_ gene) is II:2. Genotype of *CFH* single nucleotide polymorphisms (snps) targeting the *CFH-H3* risk (TGTGT) haplotype (c.1–331C>T, rs3753394; c.184G>A, p.V62I, rs800292; c.1204T>C, p.Y402H, rs1061170; c.2016A>G, p.Q672Q, rs3753396; c.2808G>T, p.E936D, rs1065489) and the *CD46 *snp (rs7144, c.*897 T>C) targeting the *CD46*_GGAAC_ risk haplotype are reported with a yellow square and in red, respectively. **(B)** MLPA analysis over the *CFH-CFHR* region in proband’s relatives shows three different patterns. Patient (II:1) exhibits one copy of *CFH* exons 22 and 23 and 3 copies of *CFHR1* exons 5 and 6. His brother (II:2) exhibits a large heterozygous deletion from *CFH* exon 22 to *CFHR1* intron 3, consistent with the presence of hybrid *CFH_1-21_-CFHR1*_5-6_ gene. The mother (I:2) has a normal copy number. These results suggest that the patient and his brother inherited the hybrid *CFH_1-21_::CFHR1_5-6_* gene from their father and evidenced the presence of a *de novo CFHR3-CFHR1* duplication only in the patient.

***CFH::CFHR1* hybrid genes**: Six unrelated patients (#1; #2; #3; #4; #5; #6) shared a similar MLPA pattern in which probes showed one copy of exon 23 (n=1) or exons 22 and 23 (n=4) or exon 21-23 (n=1) of *CFH* and a gain of exon 6 or exons 5-6 or exons 4-6 of *CFHR1*, respectively (**Table 2** and **Figure 2A**). In patients #1, #2, #3 and #4 the abnormal MLPA pattern is consistent with a deletion giving rise to *CFH_1-21_::CFHR1_5-6_* hybrid gene, described for the first time in a family in UK (29). Notably, patient #1 also exhibited an extra copy of both *CFHR3* and *CFHR1*. CNV analysis of his relatives found the *CFH_1-21_::CFHR1_5-6_* hybrid gene in the patient and in his healthy brother – with the latter lacking the extra copy of *CFHR3* and *CFHR1–* and a normal copy number in his healthy mother. Biological samples from the father were not available. Nonetheless, these results suggest that the patient inherited the hybrid *CFH_1-21_::CFHR1_5-6_* gene from his father and evidenced the presence of a *de novo CFHR3-CFHR1* duplication (**Figure 3**). In patients #2, #3 and #4, the breakpoints of the *CFH_1-21_::CFHR1_5-6_* hybrid gene were located to different genomic positions within intron 21 (#2, #3 chr1: 196712875-196797547; #4 chr1: 196712997-196797845).

Patient #4 is a familial aHUS case with an affected first cousin (III-4; **Supplementary Figure 1**). MLPA studies revealed the *CFH_1-21_::CFHR1_5-6_* hybrid gene in both patients and in three unaffected family members (26).

In patient #5 we identified the same *CFH_1-22_::CFHR1_6_* genomic rearrangement described by Maga et al., which encodes the same fusion protein as the *CFH_1-21_::CFHR1_5-6_* hybrid gene (FH_1-19_::FHR-1_5_ and FH_1-18_::FHR-1_4-5_ respectively, **Figure 2B**) (30).

**Figure 4:**
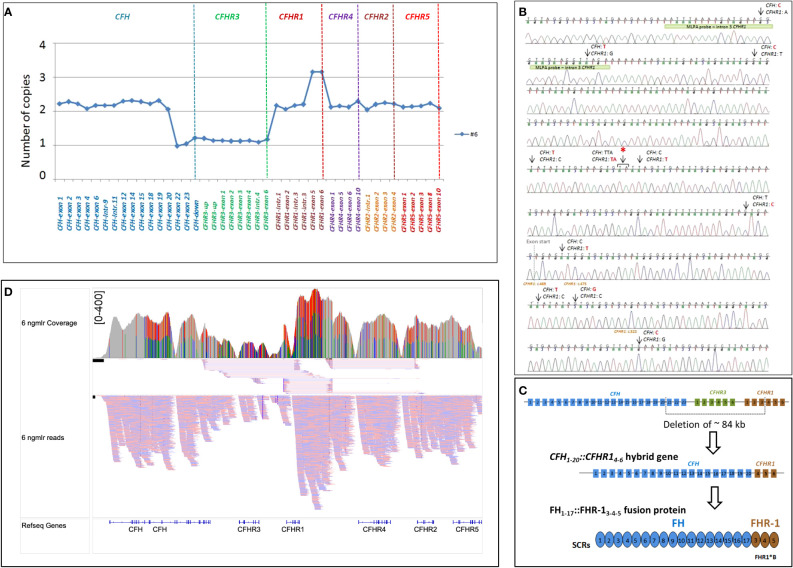
Identification of *CFH_1-20_::CFHR1_4-6_* hybrid gene in patient #6. **(A)** The abnormal MLPA pattern found in patient #6 involves *CFH*, *CFHR3* and *CFHR1* genes. It results in: 1) loss of one copy of exons 22-23 of *CFH; 2)* loss of 1 copy of the entire *CFHR3* gene; 3) 2 copies of intron 1-2 and 3 of *CFHR1*; 4) 3 copies of exon 5 and 6 of *CFHR1.* **(B)** Electropherogram including the sequence of the genomic breakpoint. Arrows indicate the nucleotide differences between *CFH* and *CFHR1.* The first part of the sequence corresponded to *CFH;* the red asterisk indicates the genomic position where the intron 4 *CFHR1* sequence started. Four nucleotide differences were found in exon 4 *CFHR1:* c.469, c.475, c.523 (indicated in brown) and c.588 (not reported). Three of them led to three FHR-1 amino acid changes (p.Tyr157-Val159-Gln175, respectively) and are characteristic of the basic isoform of CFHR1 (CFHR1*B). The green bar highlights the target sequence of the “*CFHR1*-intron 3” probe, located in intron 3 of *CFHR1 (*194 nucleotides before exon 4), upstream of the breakpoint region, explaining the 2 copies identified by this probe. **(C)** Representation of ~84 kb deletion involving *CFH*, *CFHR3* and *CFHR1*, resulting in the generation of *CFH_1-20_::CFHR1_4-6_* hybrid gene that encodes the FH_1-17_::FHR1_3-5_ fusion protein. **(D)** Screenshot from IGV (Integrative Genomics Viewer) showing reads from SMRT sequencing. SMRT sequencing identified both *CFH_1-20_::CFHR1_4-6_* hybrid gene and the *CFHR1* duplication. Misaligned reads in the *CFHR1-CFHR4* intragenic region and the *CFHR2* are also shown.

As shown in **Figure 4**, in patient #6, a large deletion extending from intron 20 of *CFH* to intron 3 of *CFHR1* was identified by MLPA and long PCR followed by Sanger sequencing (**Figures 4A, B**), indicating a novel *CFH_1-20_::CFHR1_4-6_* hybrid gene, which resulted in a FH_1-17_::FHR-1_3-5_ protein (**Figure 4C**). Since SCR3 and SCR4 of FHR-1 are identical to SCR18 and SCR19 of FH, the FH_1-17_::FHR-1_3-5_ protein is indistinguishable from FH_1-19_::FHR-1_5_ and FH_1-18_::FHR-1_4-5_ (**Figure 2B**). The genomic breakpoints were mapped between chr1: 196712504 (intron 20 of *CFH*) and chr1: 196797138 (intron 3 of *CFHR1*). The identification of the *CFH_1-20_::CFHR1_4-6_* hybrid gene, confirmed also by SMRT sequencing (**Figure 4D**), did not fully clarify the abnormal *CFHR1* MLPA pattern, characterized by the presence of 2 copies of intron 1, exon 2 and intron 3 and 3 copies of exons 5-6 of *CFHR1.* Together, these data indicated the presence of an extra copy of *CFHR1.*

Results of WB analysis of patients’ plasma/serum are shown in **Supplementary Figure 2A**.

**Reverse *CFHR1::CFH* genes**: In patients #7, #8 and #9, the exon 6 of *CFHR1* was replaced by the exon 23 of *CFH*, generating a reverse *CFHR1_1-5_::CFH_23_* hybrid gene, in addition to the normal *CFHR1* (**Figure 2C**). As we showed in a previously published study, we also observed an extra copy of *CFHR3* in patient #8 (27). Both the *CFHR1_1-5_::CFH_23_* hybrid and the extra *CFHR3* genes were transmitted to his progeny: his daughter developed aHUS, while his son was an unaffected carrier (**Supplementary Figure 1**). We previously showed that both these abnormalities were the result of a genomic duplication (27).

Patients #7 and #9 showed 3 copies of *CFH-*exon 23 and *CFH*-downstream probes and one copy of the *CFHR1*-exon 6 probe, consistent with the reverse *CFHR1_1-5_::CFH_23_* hybrid gene, but they had a normal *CFHR3* and *CFHR1* copy number. The presence in both patients of the heterozygous rs7542235 snp that tags the *CFHR1–CFHR3* deletion suggests the presence of the reverse *CFHR1_1-5_::CFH_23_* hybrid gene, with a normal *CFHR1* copy and the extra *CFHR3* copy on one allele and the common *CFHR3-CFHR1*del on the other allele (31).

In patients #10, #11 and #12, exons 5 and 6 of *CFHR1* were replaced by exons 22 and 23 of *CFH* (Reverse *CFHR1_1-4_::CFH_22-23_* hybrid gene; **Figure 2C**). Similar to patient #8, in patient #10 we also identified a third copy of *CFHR3* and 2 copies of *CFHR1*, as a result of a large genomic duplication.

The MLPA pattern of patient #11 was consistent with the extra reverse *CFHR1_1-4_::CFH_22-23_* hybrid gene, two copies of *CFHR3* and one copy of *CFHR1* (**Figure 2C**). The pedigree study showed that the proband inherited the reverse *CFHR1_1-4_::CFH_22-23_* hybrid gene from his mother and the common *CFHR3-CFHR1*del from his father (**Supplementary Figure 1**), indicating that the allele with the reverse hybrid carries a large genomic duplication, involving *CFHR3*, as reported in patient #10.

A similar but not identical MLPA pattern (not involving the probe located downstream of *CFH*; **Figure 2C**) was observed in patient #12. In both patients #11 and #12, Sanger sequencing placed the genomic breakpoints between intron 4 of *CFHR1* and intron 21 of *CFH*, confirming a reverse *CFHR1-CFH* gene, but the genomic locations were different (#11: chr1: 196799104-196714588; #12: chr1: 196797930-196713442). Patient #12 did not carry the rs7542235 snp linked to the *CFHR3*-*CFHR1* deletion, which does not indicate the presence of a deletion on the other allele, unlike patient #11. Altogether these data show that the reverse hybrid in patients #11 and #12 could derive from different genomic rearrangements. A schematic representation of the reverse FHR-1::FH proteins is shown in **Figure 2D**. Results of WB analysis of patients’ plasma/serum are shown in **Supplementary Figure 2B**.

**Figure 5:**
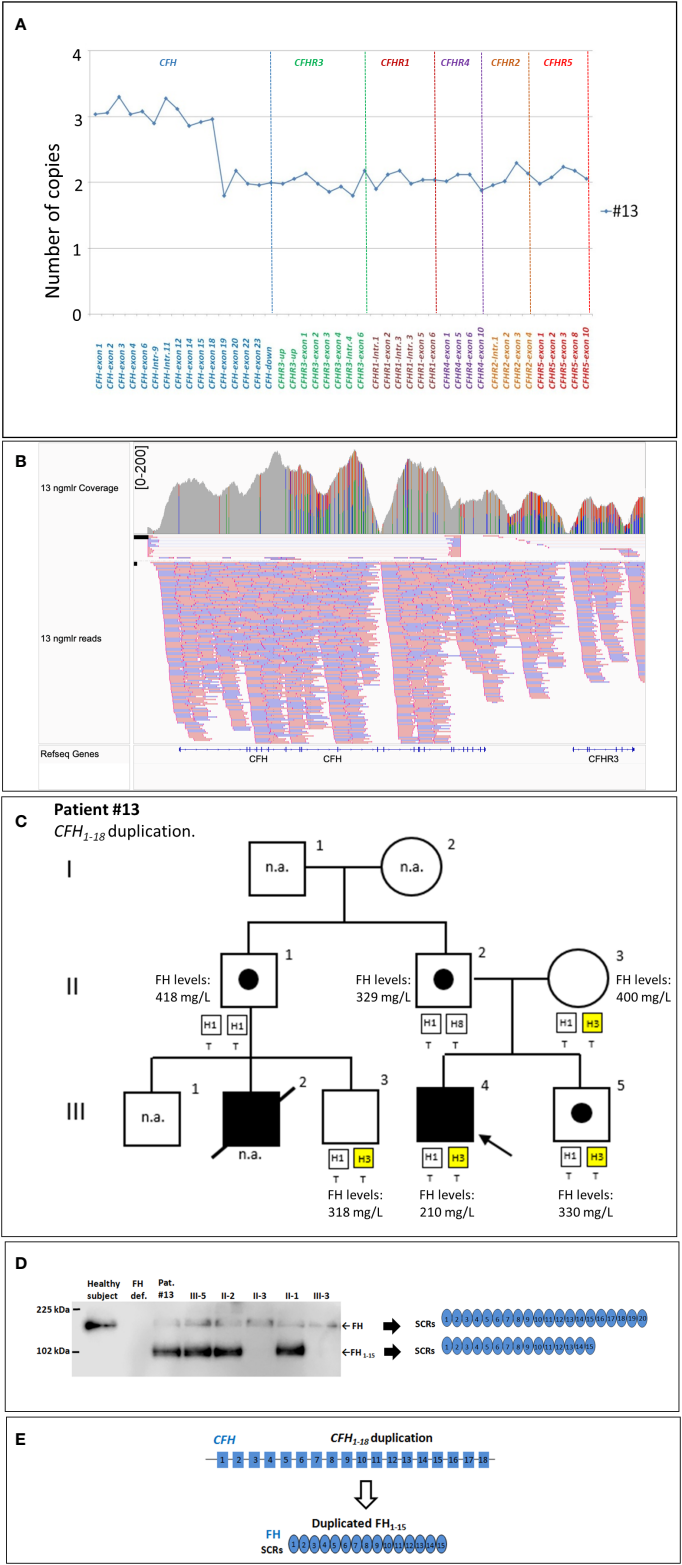
The *CFH*_1-18_ duplication identified in patient #13. **(A)** MLPA pattern showing 3 copies of *CFH* until exon 18 and 2 normal copies in the remaining *CFH*, *CFHR* exons. **(B)** Screenshot from IGV (Integrative Genomics Viewer) showing reads from SMRT sequencing of patient #13, carrying a tandem *CFH*_1-18_ duplication. Reads originating from across the breakpoint were mapped as chimeric alignments (split-reads) with the second part of the read mapped upstream of the first part (and vice versa for the reverse reads). **(C)** Pedigree of patient #13 and FH levels: the *CFH_1-18_* was inherited from the unaffected father and was also found in both his healthy brother (III-5) and in unaffected paternal uncle (II-1). FH levels resulted in the normal range in all tested samples (n.r.: ≥193 mg/L) although in the proband’s sample were lower (210 mg/L) than in the other relatives. **(D)** Western Blot (WB) to detect FH was performed using a monoclonal anti-human FH antibody (OX-23, LSBio), under non-reducing conditions, using sample from the proband (III-4), his available relatives, a patient with FH deficiency (negative control) and a healthy control with normal FH (positive control). The presence of a band with a MW (around 100 kDa) lower than normal FH in all carriers of the *CFH*_1-18_ duplication indicates that a short FH, likely missing the C-terminal domains [FH_1-15_; **(E)**], is secreted. n.a., not available.

**New genomic rearrangements:** We identified a 98 kb tandem duplication in *CFH* extending from exon 1 to exon 18 (chr1: 196611131-196708834) in a single case affected by a primary form of aHUS (#13; **Figures 5A, B** **)**. The duplication was inherited from the unaffected father (II-2) and was also found in his healthy brother (III-5) and in an unaffected paternal uncle (II-1; **Figure 5C**). Their family history shows that a paternal cousin (III-2; son of II-1) had recurrent aHUS and died at 10 years of age. FH levels in the proband and in the available relatives were normal (≥193 mg/L) although the proband had lower FH levels (210 mg/L) than his relatives (**Figure 5C**). WB using a monoclonal anti-human FH antibody and samples from all carriers of the *CFH_1-18_* duplication showed: 1) the band of normal FH protein, around 155 kDa; 2) a shorter than normal band with a MW around 100 kDa (**Figure 5D**). These results indicate that the *CFH_1-18_* duplication produces a short FH, likely composed by the first fifteen SCRs of FH (FH_1-15_) (**Figure 5E**).

**Figure 6:**
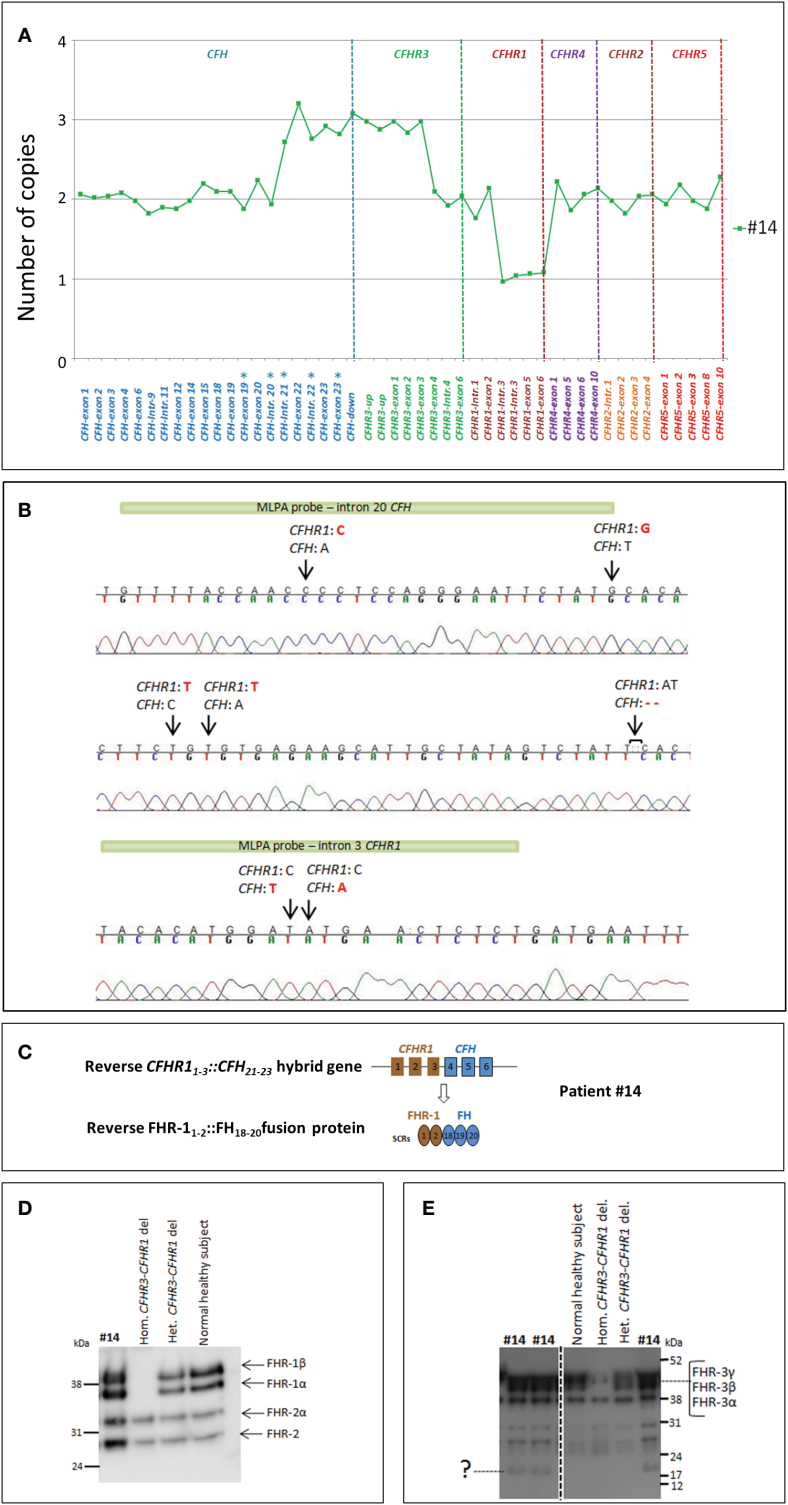
Identification of the reverse *CFHR1_1-3_::CFH_21-23_* hybrid gene in patient #14. **(A)** The results of MLPA show in patient #14 three copies of both *CFH* exons 21-22-23 and *CFHR3* exons 1-2-3, 2 copies of *CFHR1*, one of them lacking exons 4-5-6. These data suggest the presence of a reverse *CFHR1_1-3_::CFH_21-23_* hybrid with a partial duplication of *CFHR3*_1-3._ The analysis has been performed with the SALSA MLPA P236-*CFH*-region Kit (MRC Holland) implemented with homemade probes (indicated by asterisks) analyzed in a separate assay and covering the last exons and introns of the *CFH* gene (27). **(B)** Sequence of the genomic breakpoint of the *CFHR1_1-3_::CFH_21-23_* hybrid gene, mapped between chr1:196796490 (intron 3 of *CFHR1*) and chr1:196711901 (intron 20 of *CFH).* Arrows indicate the nucleotide differences between *CFH* and *CFHR1.* The green bars highlight the target sequence of “*CFH*-intron 20” probe (located 747 nucleotides before exon 21) and the “*CFHR1*-intron 3” probe (located 396 nucleotides after exon 3). **(C)** Representation of reverse *CFHR1_1-3_::CFH_21-23_* and the corresponding FHR-1_1-2_::FH_18-19-20_ fusion protein. **(D, E)** To investigate the effect of *CFHR3_1-3_* duplication at protein level, we performed a WB analysis, under non-reducing conditions, using both an anti-FHR-1-2-5 monoclonal antibody and a FHR-3 polyclonal antiserum. FHR-1 staining showed FHR-1 bands at the same MW of the normal healthy control **(D)**. Staining of FHR-3 showed both the bands corresponding to the three normal glycosylated isoforms of FHR-3 and a faint band at low MW consistent with a short FHR-3_1-2_ **(E)**.

An additional new SV was observed in patient #14, characterized by 3 copies of *CFH* intron 21-exons 22-23 and *CFHR3* exons 1-2-3, with the concomitant presence of 2 copies of *CFHR1*, one of them lacking exons 4-5-6 (**Figure 6A**). PCR, using a forward primer located in intron 2 of *CFHR1* and a reverse primer located in intron 21 of *CFH*, and Sanger sequencing revealed the presence of a *CFHR1_1-3_::CFH_21-23_* hybrid gene, likely resulting in a reverse FHR-1_1-2_::FH_18-20_ (**Figures 6B, C**). Western Blot analysis using an anti-FHR-1-2-5 antibody did not discriminate between the wt FHR1 and the reverse hybrid FHR-1_1-2_::FH_18-20_ (same molecular weight, **Figure 6D**). Analysis using an anti-FHR-3 antiserum revealed in the serum from patient #14: 1) three bands corresponding to normal glycosylated isoforms of FHR-3 (**Figure 6E** and **Supplementary Figure 2C**); 2) a single band at lower MW (around 15 kDa; **Figure 6E**) compatible with a shorter FHR-3_1-2_ protein.

### Primary aHUS group B

***CFHR3_1-5_::CFHR4_10_* hybrid gene and *CFHR1- CFHR4* duplication:** This group was characterized by uncommon genomic rearrangements involving only *CFHR* genes (**Table 2**).

In five patients (#15, #16, #17, #18, and #19) we identified the *CFHR3_1-5_::CFHR4_10_* hybrid gene that we previously described in C3G (17). Among these, case #16 also carried the polymorphic *CFHR3-CFHR1*del, leading to the lack of *CFHR1*.

In this group the only available pedigree was that of patient #15. MLPA studies revealed the presence of the *CFHR3_1-5_::CFHR4_10_* hybrid gene in his healthy mother, too (**Supplementary Figure 1**). Results of WB analysis of patients’ plasma/serum are shown in **Supplementary Figure 2D**.

Finally, four copies of both the *CFHR1* and *CFHR4* genes were observed in patient #20, a previously described case of aHUS concomitant to primary hyperoxaluria due to *GRPHR* gene abnormalities (**Supplementary Figure 1**) (28).

### Genetic and serum abnormalities in primary aHUS

To better characterize patients with uncommon SVs, we also evaluated the presence of LPVs in complement genes and or/anti-FH antibodies (anti-FHs).

We observed that group B had a higher prevalence of concomitant complement abnormalities (4/6) compared to group A (2/14) even though the difference was not statistically significant (p-value= 0.04).

In detail, only two patients out of 14 (14%; patients #3 and #4) of group A, both carrying the *CFH_1-21_::CFHR1_5-6_* hybrid gene, had additional complement abnormalities (*CFH* p. R1210C and *CFI* c.1429+1G>C LPVs, respectively).

In group B, three of six patients (50%; patients #15, #16 and #18), all carrying the *CFHR3_1-5_::CFHR4_10_* hybrid gene, also had concomitant LPVs (#15: *C3* p.D1115H and #18: *CD46* c.286+2T>G, respectively) or anti-FHs (patient #16, with the *CFHR3_1-5_::CFHR4_10_* hybrid gene on one allele and the *CFHR3-CFHR1*del on the other allele) (**Table 2**).

To evaluate whether the presence of SVs impaired circulating factor H (FH) levels we tested serum/plasma FH concentrations in all patients carrying uncommon SVs, for whom samples were available. Only patient #10, with the reverse *CFHR1_1-4_::CFH_22-23_* hybrid gene (group A), had FH levels that were slightly lower than normal (182 mg/L; n.v.≥ 193 mg/L), indicating that *CFH-CFHR* SVs did not substantially impact FH levels (**Table 2**).

Finally, to evaluate whether in our cohort of primary aHUS patients, the complete *CFHR1* deficiency was associated with the presence of anti-FHs, patients carrying the homozygous *CFHR3-CFHR1*del or the combined *CFHR3-CFHR1*del and *CFHR1-CFHR4*del were tested for anti-FHs, when samples were available. We found that 26 out of 39 tested patients had anti-FHs (67%), consistently with the already known correlation between *CFHR1* deficiency and development of anti-FHs in patients with aHUS (3, 18).

### Incomplete penetrance of rare structural variants

Among the nine studied pedigrees, we found 17 asymptomatic relatives carrying rearrangements in *CFH* or *CFHR* genes, indicating that SVs are associated with an incomplete penetrance of the phenotype aHUS (11 affected/28 carriers; 39%).

As reported above, in patients #3, #4, #15, #16 and #18 we identified additional complement abnormalities. Samples from relatives were available for patients #4 and #15 only. In the pedigree of patient #4, the *CFI* LPV (c.1429+1G>C) was found in the proband (IV-3), but also in her healthy mother (III-1) and in the younger sister (IV-4), who do not carry the *CFH_1-21_::CFHR1_5-6_* hybrid gene (**Supplementary Figure 1**). These results suggested that the concomitant presence of SVs and LPVs synergized in determining disease development in the proband. Accordingly, her father (III-2) and the older sister (IV-2) carried only the *CFH_1-21_::CFHR1_5-6_* hybrid gene and did not have aHUS. However, the *CFH_1-21_::CFHR1_5-6_* hybrid gene but not the *CFI* LPV (c.1429+1G>C), were also found in a healthy grand-uncle of the proband (II-3) and his daughter (III-4), who developed ESRD after an episode of aHUS. These findings indicate that in this arm of the pedigree, other risk factors synergized with the *CFH_1-21_::CFHR1_5-6_* hybrid to the final phenotype. As shown in **Supplementary Figure 1**, the affected subject in this arm (III-4) is homozygous for the *CD46_GGAAC_* risk haplotypes, whereas her unaffected father is heterozygous.

Incomplete penetrance was also observed in the pedigree of patient #15 carrying the *CFHR3_1-5_::CFHR4_10_* hybrid gene, the *C3* LPV (p.D1115H), and the *CFH-H3* risk haplotype, all inherited from the unaffected mother (**Supplemantary Figure 1**).

### Clinical data of patients with primary aHUS carrying rare SVs

In group A, infectious triggers were reported in all patients with available data (7/7) and the median age at disease onset was 6.5 years (IQR, 1-25.75). Two out of 14 patients were treated with eculizumab: one of them (patient #9) underwent full remission with complete recovery of renal function, the other (patient #7) had an aHUS relapse in the kidney graft. Eculizumab treatment enabled stable normalization of hematological parameters and partial recovery of graft function, but thereafter the patient lost the graft due to chronic rejection. In contrast, 11 out of 12 patients in this group who did not receive eculizumab (either because disease onset antedated anti-complement therapy or the drug was not available) did not recover from the acute episode and developed end-stage renal disease (ESRD; **Table 3**).

**Table 3:** Demographic and clinical findings of aHUS patients carrying uncommon *CFH-CFHR* SVs.

Pat.	Sex	Age	Trigger	Outcome	Therapy	Eculizumab (Yes/no)	Relapses (Yes/No)	N° of Tx	Eculizumab prophylaxis Tx1	Relapses Tx1	Eculizumab treatment Tx1	Outcome of Tx1	Eculizumab prophylaxis Tx2	Relapses Tx2	Eculizumab treatment Tx2	Outcome of Tx2
**Pat.**	**Sex**	**Age**	**Trigger**	**Outcome**	**Therapy**	**Eculizumab (Yes/no)**	**Relapses (Yes/No)**	**N° of Tx**	**Eculizumab prophylaxis Tx1**	**Relapses Tx1**	**Eculizumab treatment Tx1**	**Outcome of Tx1**	**Eculizumab prophylaxis Tx2**	**Relapses Tx2**	**Eculizumab treatment Tx2**	**Outcome of Tx2**
Primary aHUS - Group A																
#1	M	1	Upper resp tract infection	ESRD	Antihypertensive, steroids, BT, PI	No	Yes	0	-	-	-	-	-	-	-	-
#2	F	1	na	ESRD	Antihypertensive	No	na	1	No	Yes	No	ESRD	-	-	-	-
#3	M	1	Viral infection	ESRD	BT, Igs, PI, PEX	No	Yes	0	-	-	-	-	-	-	-	-
#4	F	0.5	Upper resp tract infection	Recovery renal fx	PI, BT, PEX	No	No	0	-	-	-	-	-	-	-	-
#5	F	0.4	gastroenteritis	ESRD	BT	No	na	2	No	Yes	No	ESRD	Yes	No	No	Normal renal fx
#6	F	21	Na	ESRD	na	No	na	1	No	Yes	No	ESRD	-	-	-	-
#7	M	3	gastroenteritis	ESRD	na	No	na	1	No	Yes	Yes	ESRD ^a^	-	-	-	-
#8	M	49	Upper resp tract infection	ESRD	BT, PI, PEX	No	na	1	Yes	No	na	Normal renal fx	-	-	-	-
#9	M	25	na	Recovery renal fx	BT, PEX	Yes	No	0	-	-	-	-	-	-	-	-
#10	F	1	na	ESRD	Antihypertensive, PEX	No	na	1	No	No	na	ESRD	-	-	-	-
#11	M	48	na	ESRD	PEX, hemodialysis	Yes ^b^	No	0	-	-	-	-	-	-	-	-
#12	F	26	na	ESRD	BT, PI, PEX	No	No	2	No	na	No	ESRD	No	Yes	No	ESRD
#13	M	10	flu-like	ESRD	PI	No	na	1	Yes	No	na	Normal renal fx	-	-	-	-
#14	M	2	na	ESRD	na	No	na	na	-	-	-	-	-	-	-	-
Primary aHUS - Group B																
#15	M	1	Upper resp tract infection	ESRD	PEX	No	Yes	2	No	Yes	No	ESRD	No	Yes	No	ESRD
#16	F	11	gastroenteritis	Recovery renal fx	BT, PI, PEX	No	na	0	-	-	-	-	-	-	-	-
#17	M	2	Upper resp tract infection	Recovery renal fx	BT, PI, PEX	No	No	0	-	-	-	-	-	-	-	-
#18	F	2	Upper resp tract infection	Recovery renal fx	BT, PI, PEX	No	Yes	0	-	-	-	-	-	-	-	-
#19	F	4	na	Na	na	No	na	0	-	-	-	-	-	-	-	-
#20	M	0.5	gastroenteritis	Recovery renal fx	BT, PI	No	No	0	-	-	-	-	-	-	-	-
Secondary aHUS																
#21	F	48	Malignant hypertension	ESRD	Antihypertensive	No	na	0	-	-	-	-	-	-	-	-
#22	F	22	Severe hypertension	Partial recovery renal fx	BT, PI	Yes	No	0	-	-	-	-	-	-	-	-

PI, plasma infusion; BT, blood transfusion; PEX, plasma exchange; Igs, immunoglobulins; ESRD, end-stage renal disease; fx, function; tx, transplant;.^a^ ESRD 8 days after transplant for chronic rejection; ^b^After 21 PEX; na, not available.

Atypical HUS relapses occurred in 6/7 grafts without eculizumab prophylaxis and in 0/3 grafts with eculizumab prophylaxis.

In group B, infectious triggers were associated with aHUS onset in 5/5 cases with available data, and the median age of disease onset was 2 years (IQR, 1.5-7.5).

Disease was less severe in group B than in group A. Indeed, 4 out of 5 patients in this group achieved complete remission without eculizumab treatment (p=0.0099, vs group A no eculizumab). No clinical data are available for patient #19.

One group B patient (patient #15) lost two kidney grafts for aHUS recurrence; he did not receive eculizumab (**Table 3**).

### Rare structural variants and clinical data of patients with secondary aHUS

**Figure 7:**
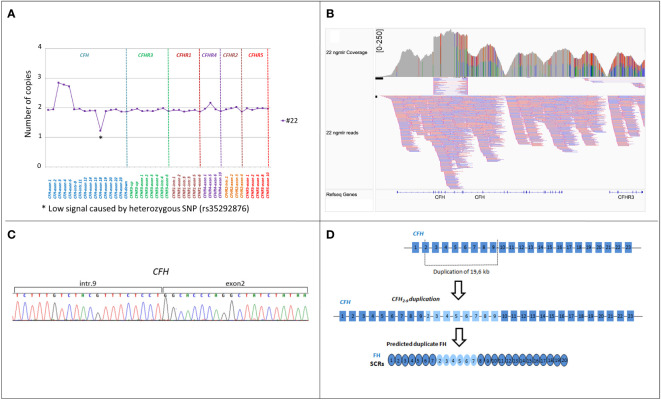
Identification of *CFH_2-9_* duplication in patient #22. **(A)** MLPA pattern in patient #22 shows a high signal on the exon 3, exon 4 and exon 6-*CFH* probes consistent with a heterozygous duplication. **(B)** Screenshot from IGV (Integrative Genomics Viewer) showing reads from SMRT sequencing. SMRT sequencing identified *CFH_2-9_* duplication. **(C)** Electropherogram of the genomic breakpoint. The first part of the sequence corresponds to intron 9 of the *CFH* and the second part is the sequence of *CFH* exon 2. **(D)** Representation of 19,6 kb internal duplication of the *CFH* and the predicted resulting FH protein consisting of 26 SCRs.

Two cases of secondary aHUS, associated with malignant and severe hypertension, respectively, had uncommon **SV** (#21; #22; **Table 2**). One of them carried the hybrid *CFHR3_1-5_::CFHR4_10_* gene, previously described in a patient with DDD (17) while the other patient had an internal duplication in *CFH*, extending from part of exon 2 to exon 9 (**Figure 7A**). Direct sequencing of a long-PCR product and SMRT sequencing allowed us to map the duplication within *CFH* (chr1:196642182-196661791; **Figures 7B, C**) with partial intron 9 sequence followed by part of exon 2 sequence. *In silico* analysis (Genscan) predicted that the *CFH*_2-9_ duplication may generate a longer *CFH* gene, characterized by the first nine exons of *CFH*, followed by exon 3 of *CFH* (**Figure 7D**).

In both patients no LPVs in complement genes were identified (**Table 2**).

The outcome of the patient with the hybrid *CFHR3_1-5_::CFHR4_10_* gene (#21) was unfavorable as she developed ESRD (no eculizumab treatment; **Table 3**). The patient carrying the internal duplication of *CFH* was treated with eculizumab, which partially improved renal function and hematological parameters at one month after disease onset (**Table 3**).

## Discussion

In the present study, through a retrospective analysis of a large cohort of patients affected by either primary or secondary forms of aHUS, we documented that: I) the prevalence of the homozygous *CFHR3-CFHR1*del is higher in primary aHUS than in secondary aHUS; II) uncommon SVs in *CFH* and *CFHR* genes are more frequent in primary aHUS than in secondary forms; III) the disease penetrance of aHUS in carriers of rare *CFH-CFHR* SVs is incomplete; IV) these genomic abnormalities occur often in combination with other complement abnormalities; V) the prognosis for rare SV carriers is strongly related to the specific abnormality.

Among common SVs, we observed an enrichment of the homozygous *CFHR3-CFHR1*del in primary aHUS and confirmed previous data in the literature about the association between this common SV and the development of anti-FH autoantibodies, an acquired driver reported in 10% of aHUS patients (3, 18). In contrast, the finding that in secondary aHUS patients the prevalence of the homozygous *CFHR3-CFHR1*del was comparable to controls does not support the hypothesis that this deletion plays a role in secondary aHUS. Similarly, we identified rare SVs, including duplications or hybrid genes, in a substantial fraction (8%) of patients with aHUS, but we rarely did so in secondary forms (2%).

Consistent with earlier data in literature, in our cohort uncommon SVs most frequently involved *CFH* and *CFHR1* leading to the formation of *CFH::CFHR1* hybrid genes or reverse *CFHR1::CFH* hybrid genes (16, 27, 29, 30, 32).

Specifically, we identified the *CFH_1-21_::CFHR1_5-6_* and the *CFH_1-22_::CFHR1_6_* SVs in 2% of patients with primary aHUS. Even though they formed through different rearrangements, as documented by the identification of different DNA breakpoints, hybrid *CFH::CFHR1* genes caused the loss of one copy of *CFHR3* and encoded for the same FH::FHR-1 fusion protein characterized by a FH protein in which SCR19 and 20 were substituted by the FHR-1 specific SCR4-SCR5 C-terminal residues (29, 30). Notably, the FH SCR19 is identical to FHR-1 SCR4, while FH SCR20 differs from FHR-1 SCR5 only at 2 amino acids: FH Ser1191 (which corresponds to Leu290 in FHR-1) and FH Val1197 (corresponding to Ala296 in FHR-1). Thus, the FH-FHR-1 fusion proteins encoded by the *CFH_1-21_::CFHR1_5-6_* and the *CFH_1-22_::CFHR1_6_* hybrids share the FHR-1-specific C-terminus with the Leu1191 and Ala1197 changes. As reported in published studies, the FH with 1191Leu and 1197Ala residues can also derive from a gene conversion event that is the result of the unidirectional transfer of *CFHR1* exon 6 into the *CFH* gene (33, 34). Functional studies have shown that these FH mutants have a normal regulatory activity in the fluid phase but have a limited capacity to protect cells from complement activation (33). This is also consistent with recent findings showing that the two amino acid differences that differentiate the C-terminus of FH from that of FHR-1 are responsible for distinctive interactions with C3 and sialic acid glycans (35, 36). Indeed, while the C-terminus of FH binds to proteoglycans and C3b, thus driving the recruitment of FH to cell surfaces and to the cell matrix, the C-terminus of FHR-1 strongly interacts with native C3 (nC3), C3b, iC3b and C3dg, attracting nC3 to the proximity of the cell surface. The latter acquired property of FH::FHR-1 fusion proteins, along with the loss of the capacity to bind sialic acids, causes a shift from complement regulation to complement activation on cell surfaces (35).

Another *CFH* and *CFHR1* genomic rearrangement reported in association with aHUS leads to reverse *CFHR1::CFH* genes. A *de novo CFHR1_1-4_::CFH_22-23_* gene was described in 2013 in a patient with sporadic aHUS, and in 2015 we reported a reverse *CFHR1_1-5_::CFH_23_* gene in a family with 2 affected subjects over 2 generations (27, 32). Here, we identified 5 additional patients carrying the above reverse *CFHR1::CFH* genes, which derived from different DNA breakpoints. Notably, all these genes encode the same FHR-1::FH fusion protein characterized by the FH specific C-terminus with amino acid 290Ser (which corresponds to Ser1191 in FH) and 296Val (that corresponds to Val1197 in FH) (37). The reverse FHR-1::FH hybrid, through its FHR-1 N-terminal domain forms multimers that interact with other FHR-1 and/or FHR-2 molecules, while with its FH C-terminal domain competes with FH for the binding to cell ligands, thus promoting surface restricted complement activation (16, 35). Consistently, FHR-1 isolated from heterozygous carriers of the *CFHR1_1-5_::CFH_23_* hybrid induced complement-dependent sheep erythrocytes hemolysis when added to normal human serum (27).

In this study, we have also identified a new reverse *CFHR1_1-3_::CFH_21-23_* gene including a partial duplication of the first three exons of *CFHR3* likely resulting in a FHR-1_1-2_-FH_18-20_ protein and in a short FHR3_1-2_ protein that requires further investigation. We speculate that the greater sequence similarity to FH, makes the FHR-1_1-2_-FH_18-20_ protein a stronger competitor of FH for its surface ligands, than the reverse FHR-1_1-4_::FH_20_ and FHR-1_1-3_::FH_19-20_ hybrids.

Another novel finding of this study is the association of large *CFH* gene duplications with aHUS. In a 10-year boy with primary aHUS, we identified a *CFH* tandem duplication involving a large portion of the gene, including exons 1 to 18, and encoding a shorter than normal FH (likely FH_1-15_) that lacks the five C-terminal SCRs. We speculate that the abnormal FH cannot properly bind and inhibit AP on cell surfaces while maintaining its inhibitory functions in fluid phase. In addition, in a 22-year-old woman with secondary aHUS associated with severe hypertension, we found an internal *CFH* duplication extending from part of exon 2 to exon 9 and located after *CFH* intron 9. We hypothesize that similarly to partial duplications described in other genes, this *CFH* duplication may either cause a reading frame shift in the mRNA, producing a truncated FH, or result in a longer than normal protein with conformational changes and dysfunctional activity (38–40). Consistently, *in silico* analysis of the *CFH*_2-9_ duplication predicts a longer translated FH product (**Figure 7D**).

Notably, we observed incomplete penetrance of aHUS in carriers of *CFH-CFHRs* SVs, which is consistent with earlier studies on patients with LPVs in *CFH* and other complement genes (23, 41, 42). The identification of additional rare complement gene abnormalities in 3 probands with *CFH-CFHRs* SVs is in line with the above observation. Specifically, in a pedigree with the *CFH_1-21_::CFHR1_5-6_* hybrid gene we found a *CFI* variant, predicted to alter splicing in the region encoding serine protease domain of Factor I, in the proband who developed aHUS in the first year of life, whereas this variant was absent in all unaffected carriers of the hybrid gene. However, the finding that in this family a carrier of the *CFH_1-21_::CFHR1_5-6_* alone developed aHUS, whereas there were none among the subjects with the *CFI* LPV alone, documents that the *CFH_1-21_::CFHR1_5-6_* hybrid gene is the main driver of the disease. In a patient with the *CFH_1-21_::CFHR1_5-6_* hybrid gene we also identified the FH R1210C LPV, which was previously reported as a predisposing factor to a range of pathologies, including aHUS, C3G and AMD. The FH-1210C mutant forms covalently linked complexes with human serum albumin that interfere with FH binding to surface-bound C3b (43, 44). Thus, due to the combination of the FH 1210C mutant from one allele and the hybrid FH with the 1191L and 1197A changes from the other allele, all FH molecules in this patient have a dysfunctional C-terminus, leading to defective regulation of complement on cellular surfaces. Finally, in a third patient carrying the *CFH_1-21_::CFHR1_5-6_* hybrid gene, familial studies revealed that the proband also has a *de novo* duplication involving *CFHR3* and *CFHR1* genes that was not found in unaffected relatives carrying the hybrid gene.

We found additional genetic or acquired abnormalities even more frequently in the group of patients with SVs involving only *CFHR* genes, which mostly result in the formation of a *CFHR3_1-5_::CFHR4_10_* hybrid. Indeed, 4 out of 6 patients in this group also carry either LPVs or anti-FH antibodies, which would indicate a lower pathogenic impact of *CFHR* SVs versus those involving *CFH*. The *CFHR3_1-5_::CFHR4_10_* hybrid has already been reported in association with C3G, and it has been suggested that it binds cell surface ligands and favors C3 convertase activity, but functional studies are required to clarify its pathogenic impact (17). The finding that a proband shared the *CFHR3_1-5_::CFHR4_10_* hybrid gene, a *C3* LPV and *CFH* H3 risk haplotype with their unaffected mother, underlines the complexity of aHUS, which may not manifest even in subjects with multiple genetic risk factors. It is likely that in such susceptible individuals, environmental factors or an underlying condition that activates complement or perturbs the endothelium are required to trigger the disease. This possibility is in line with the report here of two cases of aHUS secondary to chronic severe hypertension in patients who carried SVs affecting the *CFH* or *CFHRs* genes, respectively. It is known that microvascular endothelium can move to a pro-thrombotic phenotype during stress stimuli due to hypertension (45, 46). So in the above patients, the concomitance of hypertension-mediated endothelial stress injury and genetically-determined defective regulation of the complement system may irreversibly compromise the homeostatic equilibrium of the endothelium.

Our data also show relevant associations between the specific SV and disease phenotype, response to therapies and risk of recurrence after kidney transplant. Thus, *CFH::CFHR1* hybrid genes were commonly found in patients who manifested the disease in their first year of life, a finding consistent with previously published data (33). The FH::FHR-1 hybrids mimic the effect of LPV in the FH C-terminus that have been often associated with disease onset in infancy (2). Finding that in the majority of *CFH::CFHR1* carriers aHUS was triggered by infections, would suggest that in these patients dysfunctional FH could not adequately control the complement activation induced upon the first exposure to viruses or bacteria. As previously reported (37), the reverse *CFHR1::CFH* hybrid genes were associated with a later disease onset, mostly in adulthood. It is tempting to speculate that in these patients FH produced by the 2 normal copies of *CFH*, could partially counteract the competitive action of the reverse FHR-1::FH protein (27, 37).

Nonetheless, both patients with hybrid *CFH::CFHR1* genes and those with reverse *CFHR1::CFH* genes from our cohort who did not receive anti-complement therapy had an unfavorable prognosis, while those treated with eculizumab went into full remission.

At variance, the outcome in patients with *CFHR* hybrids was more favorable than in patients with *CFH* SVs, even when they did not receive eculizumab, confirming a lower pathogenic impact of *CFHR* SVs than *CFH* SVs.

Previous reports from the pre-eculizumab era documented a strong association between *CFH* genetic abnormalities and the risk of relapses after kidney transplant, almost invariably leading to graft loss (2). In recent studies the use of prophylactic eculizumab was independently associated with a reduced risk of recurrence and with longer graft survival (47, 48). Consistent with this, here we observed overall unfavorable outcomes in 8 out of 9 grafts without prophylactic eculizumab, while 3 grafts transplanted under eculizumab prophylaxis have maintained normal function.

In conclusion, this work highlights the association between aHUS and genomic rearrangements in the *CFH-CFHR* region and describes both known genomic alterations and new large aberrations, which are often hard to identify and solve. These structural variants have a different impact on risk of disease manifestation, age of onset, and severity. Not surprisingly, our findings further highlight the prevalent role of factor H genetic defects in the pathogenesis of aHUS but also propose that abnormalities in factor H-related proteins may play a role. Altogether, our data definitely support including a *CFH-CFHR* SV search in routine genetic analysis for patients with aHUS to improve prognosis and treatment approaches.

## Data availability statement

The data presented in the study are deposited in the EBI European Nucleotide Archive, accession number: PRJEB44176.

## Ethics statement

The studies involving human participants were reviewed and approved by Ethics Committee of the Azienda Sanitaria Locale, Bergamo (Italy). Written informed consent to participate in this study was provided by the participants’ legal guardian/next of kin.

## Author contributions

RP, EV, MA and MN designed research, interpreted data, and wrote the paper. EV, RP, MA, LL, CM, MB, and RD performed the research and analyzed the data. EB and MR provided detailed clinical information on patients. MN, AB and GR critically revised the manuscript. All authors contributed to the article and approved the submitted version.

## Supplementary material

Supplementary Figure 1 Analyzed pedigrees. Each proband is indicated by a black arrow. Black squares and cicles indicate affected subject. The black dots show carriers of the SV. Genotype of *CFH* single nucleotide polymorphisms (snps) targeting the *CFH-H3* risk (TGTGT) haplotype (c.1–331C>T, rs3753394; c.184G>A, p.V62I, rs800292; c.1204T>C, p.Y402H, rs1061170; c.2016A>G, p.Q672Q, rs3753396; c.2808 G>T, p.E936D, rs1065489) and the *CD46*snp (rs7144 c.*897 T>C) targeting the *CD46*_GGAAC_ risk haplotype are reported with a yellow square and in red, respectively.

Supplementary Figure 2 Western blot images. **(A)** Staining of FH detecting FH_1-18_::FHR-1_4-5_ hybrid protein in patients #1 and #3 and FH_1-17_::FHR-1_3-5_ in patient #6 that have the same MW of normal FH. Similarly, reverse FHR-1::FH hybrid proteins tested in serum from patients #7 (FHR-1_1-4_::FH_20_), #10, #11 (FHR-1_1-3_::FH_19-20_) and #14 (FHR-1_1-2_::FH_18-20_) share the same MW of normal FHR-1 **(B)**. **(C)** WB using a FHR-3 antiserum was performed to evaluate the protein pattern of patients also carrying *CFHR3* duplications (#1 and #10). The image shows in #1 and #10, bands of increased intensity compared to healthy subject and patient #11 (both carrying normal copies of *CFHR3*) and with the same MWs of the 3 normal glycosylated isoforms of FHR-3. WB analysis using the sample from patient #14, carrying the *CFHR3_1-3_* duplication was also analyzed and results show the presence of FHR-3 bands with expected MWs. A single band at lower MW (around 15kDa) compatible with a shorter FHR-3_1-2_ protein was observed in the WB reported in . **(D)** WB images showing the 3 bands of FHR-3 in patients #15, #16 and #17 (all carrying the *CFHR3_1-5_-CFHR4_10_*) indicating that the FHR3_1-4_-FHR4_9_ hybrid protein is secreted and has the same MW of normal FHR-3 since patient #16 carries the *CFHR3-CFHR1* deletion on the other allele.
