# Supplementary figures and images for: CFH and CFHR structural variants in atypical Hemolytic Uremic Syndrome: Prevalence, genomic characterization and impact on outcome
Source: Front Immunol. 2023 Jan 30;13:1011580. doi: 10.3389/fimmu.2022.1011580 (PMC9923232; doi:10.3389/fimmu.2022.1011580)

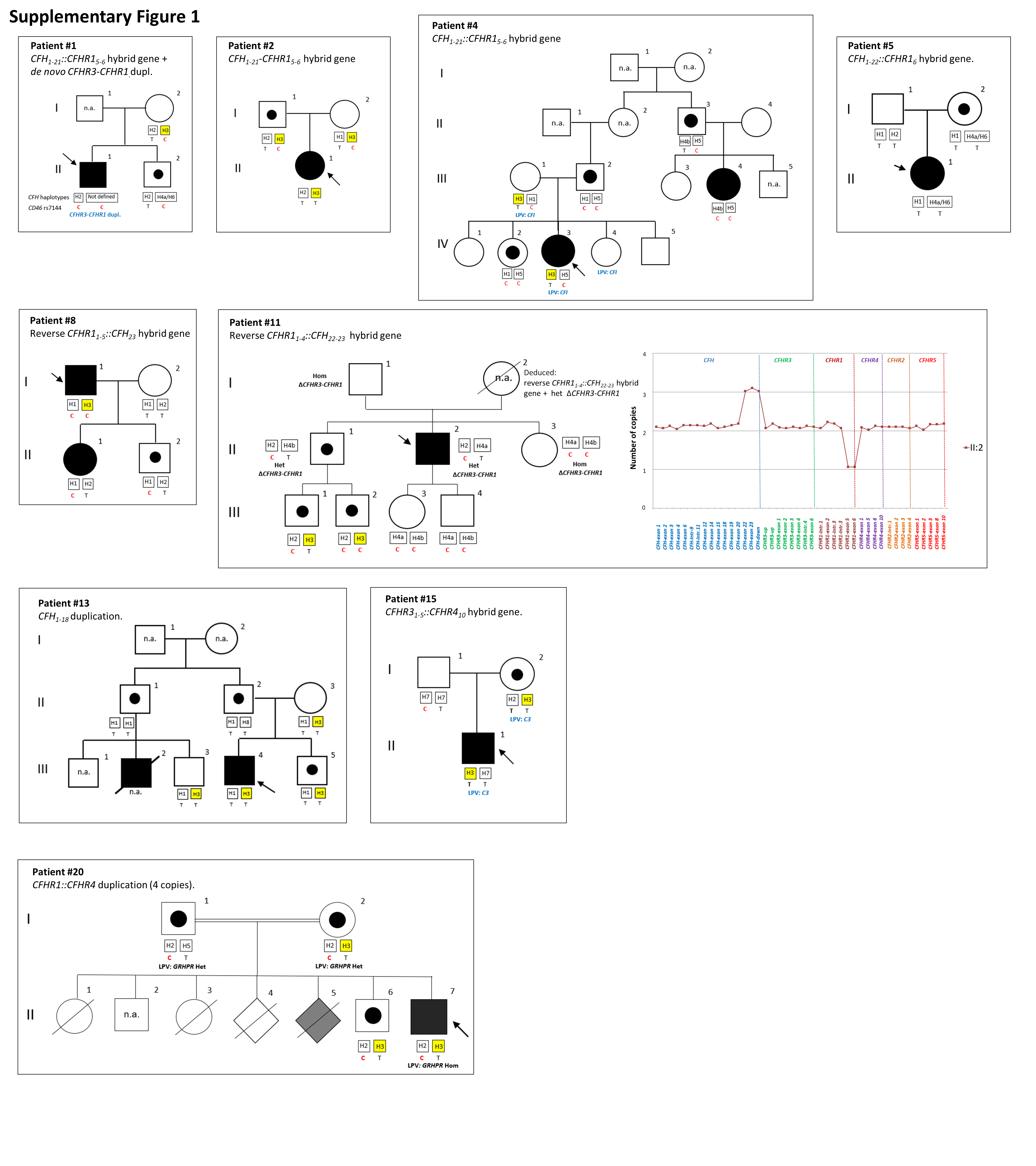

Supplement: Supplementary Figure 1 — Analyzed pedigrees. Each proband is indicated by a black arrow. Black squares and cicles indicate affected subject. The black dots show carriers of the SV. Genotype of CFH single nucleotide polymorphisms (snps) targeting the CFH-H3 risk (TGTGT) haplotype (c.1–331C>T, rs3753394; c.184G>A, p.V62I, rs800292; c.1204T>C, p.Y402H, rs1061170; c.2016A>G, p.Q672Q, rs3753396; c.2808 G>T, p.E936D, rs1065489) and the CD46snp (rs7144 c.*897 T>C) targeting the CD46 GGAAC risk haplotype are reported with a yellow square and in red, respectively. [file Image_1.tif]

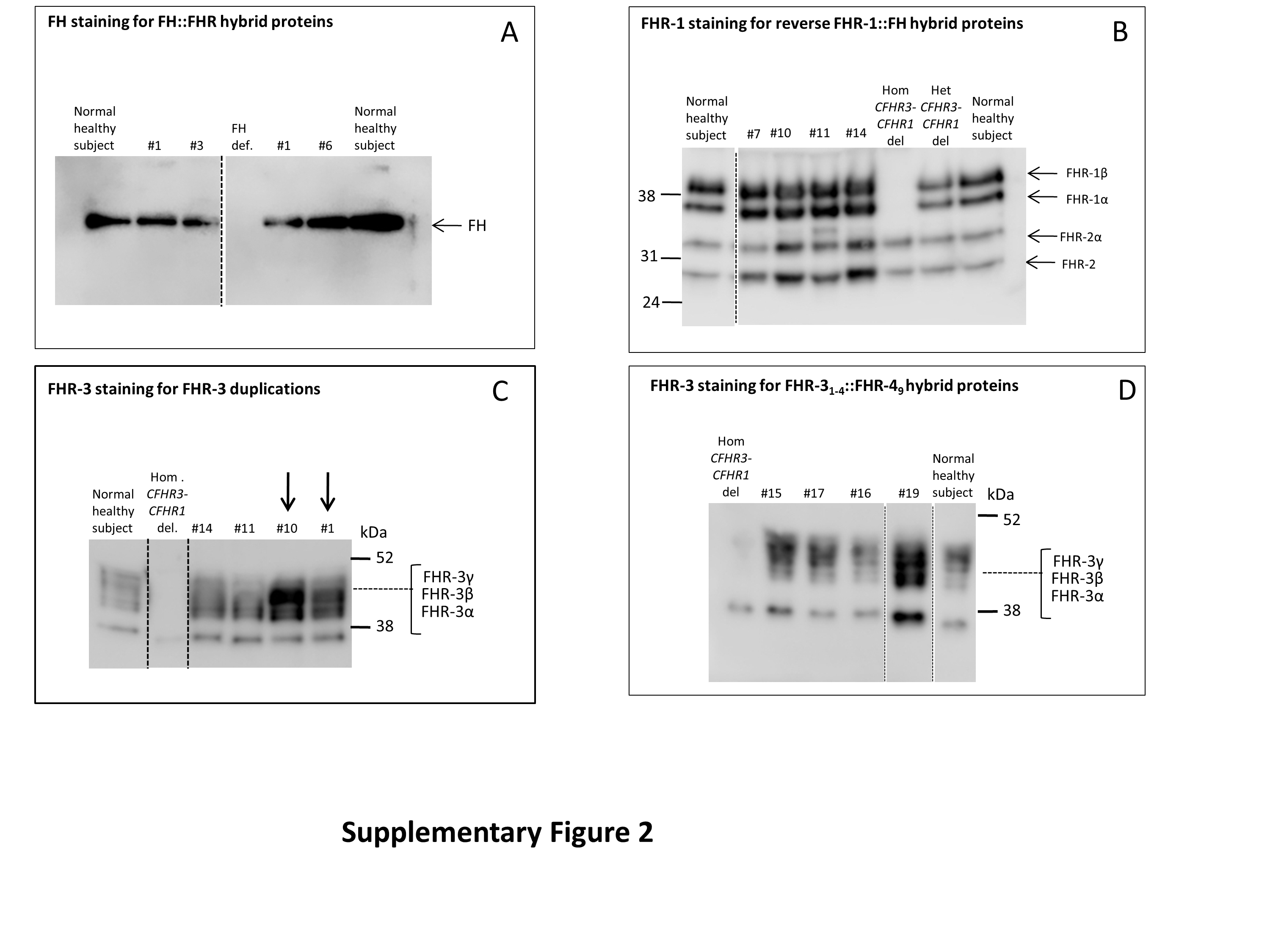

Supplement: Supplementary Figure 2 — Western blot images. (A) Staining of FH detecting FH1-18::FHR-14-5 hybrid protein in patients #1 and #3 and FH1-17::FHR-13-5 in patient #6 that have the same MW of normal FH. Similarly, reverse FHR-1::FH hybrid proteins tested in serum from patients #7 (FHR-11-4::FH20), #10, #11 (FHR-11-3::FH19-20) and #14 (FHR-11-2::FH18-20) share the same MW of normal FHR-1 (B). (C) WB using a FHR-3 antiserum was performed to evaluate the protein pattern of patients also carrying CFHR3 duplications (#1 and #10). The image shows in #1 and #10, bands of increased intensity compared to healthy subject and patient #11 (both carrying normal copies of CFHR3) and with the same MWs of the 3 normal glycosylated isoforms of FHR-3. WB analysis using the sample from patient #14, carrying the CFHR31-3 duplication was also analyzed and results show the presence of FHR-3 bands with expected MWs. A single band at lower MW (around 15kDa) compatible with a shorter FHR-31-2 protein was observed in the WB reported in . (D) WB images showing the 3 bands of FHR-3 in patients #15, #16 and #17 (all carrying the CFHR31-5-CFHR410 ) indicating that the FHR31-4-FHR49 hybrid protein is secreted and has the same MW of normal FHR-3 since patient #16 carries the CFHR3-CFHR1 deletion on the other allele. [file Image_2.tif]

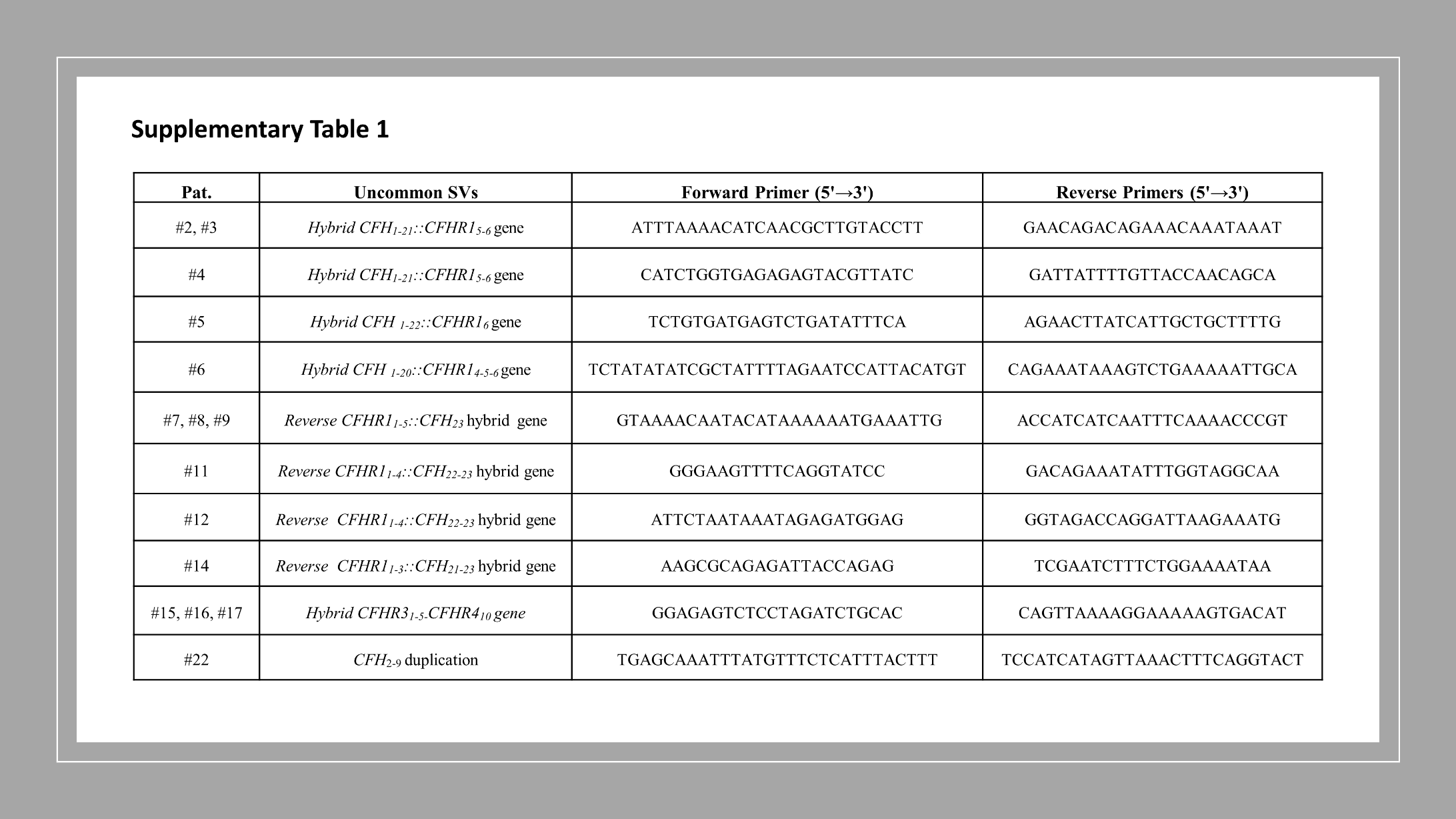

Supplement: Supplementary file 3 [file Table_1.docx]
